# Supplementary material for: Metabolic Footprint, towards Understanding Type 2 Diabetes beyond Glycemia
Source: J Clin Med. 2020 Aug 10;9(8):2588. doi: 10.3390/jcm9082588 (PMC7463676; doi:10.3390/jcm9082588)
Supplement: Supplementary file 1 [file jcm-09-02588-s001.pdf]

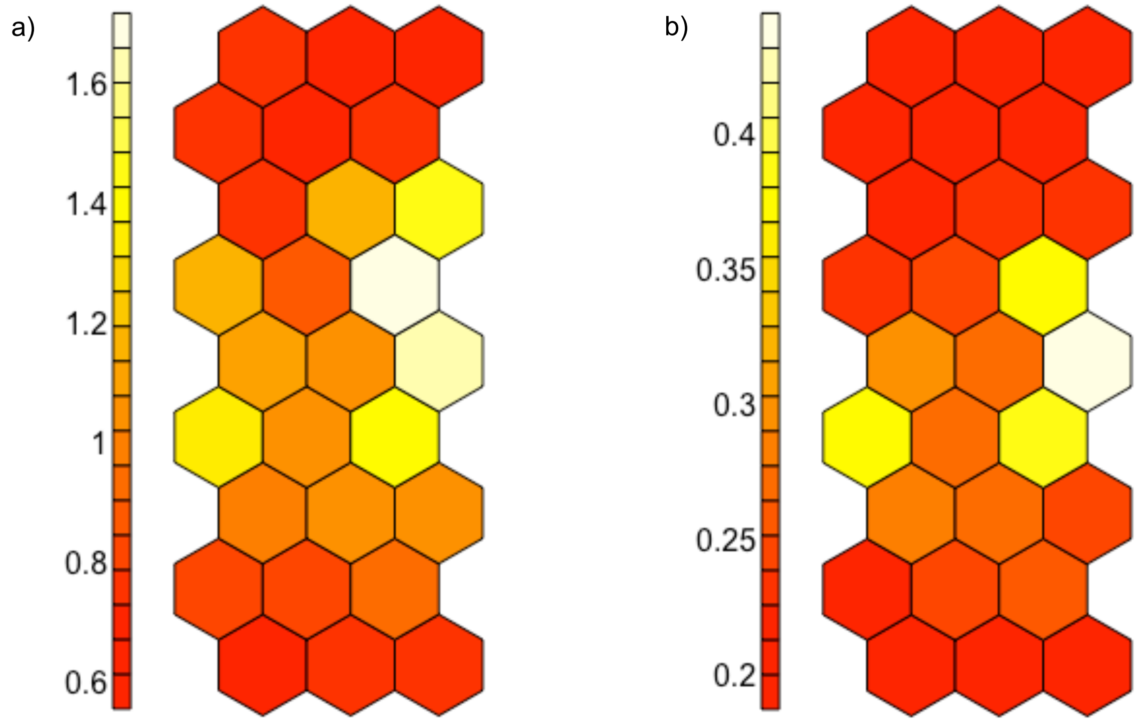

Supplementary Figure1. Self Organizing Map (SOM) neighbor distance plot (a) and quality plot (b) of the 27 SOM units.

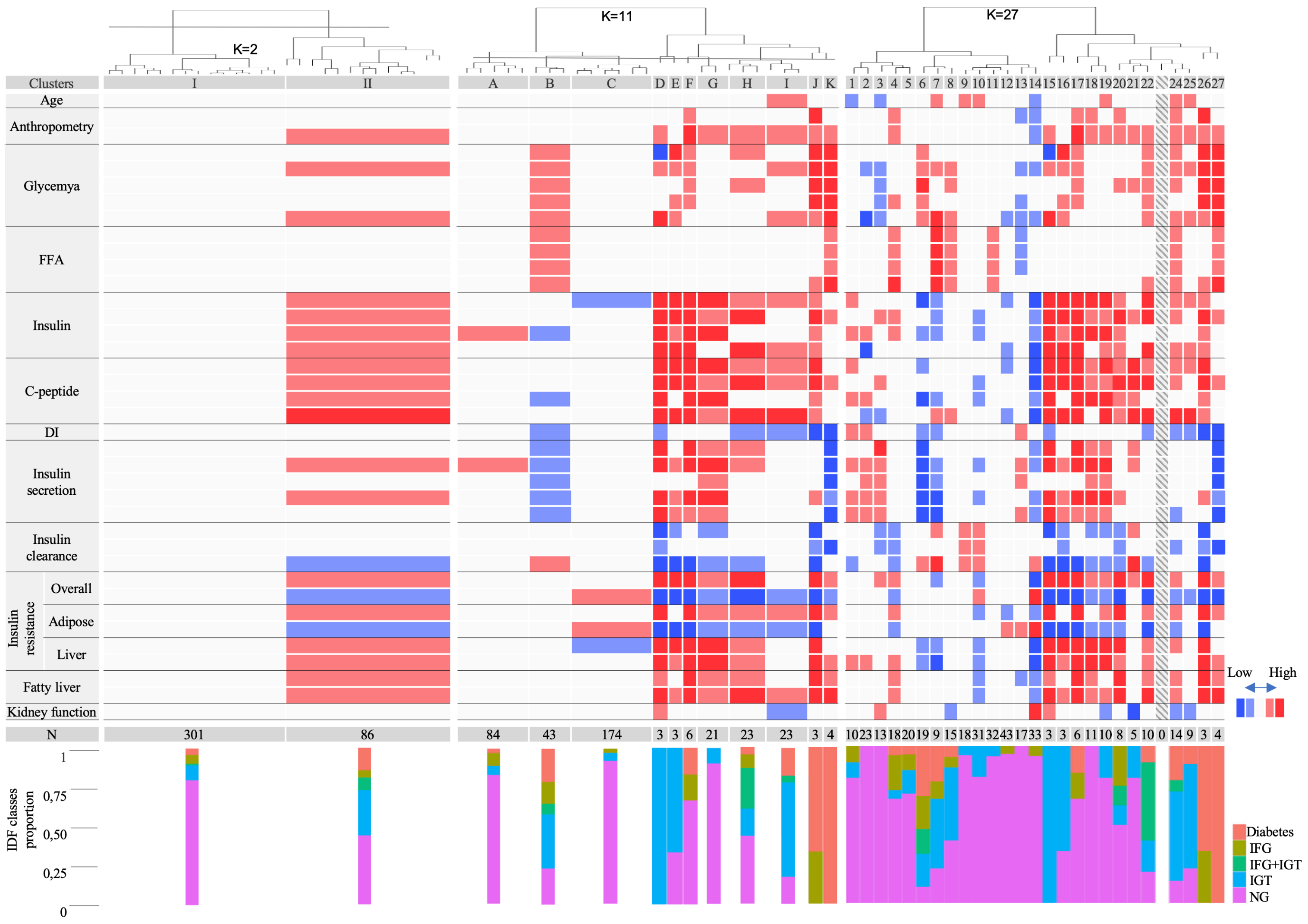

Supplementary Figure 2. Clusters profiling for the male population. Dendogram of the hierarchical clustering of the 27 Self Organizing Map (SOM) units is shown for three cut levels of the dendogram(a): K=2 (clusters I and II); K=11 (clusters A-K) and K=27 (clusters 1-27). For each level, the clusters were profiled with several parameters, herein grouped by function, and the median of each cluster was compared with the distribution of the overall population (percentiles 10,25,75,90), and are displayed with a heatmap scale (b). Number of subjects in each cluster is shown in (c). Distribution of IDF dysglycemia classes in each cluster is shown in (d). FFA, free fatty acids; DI, Disposition Index.

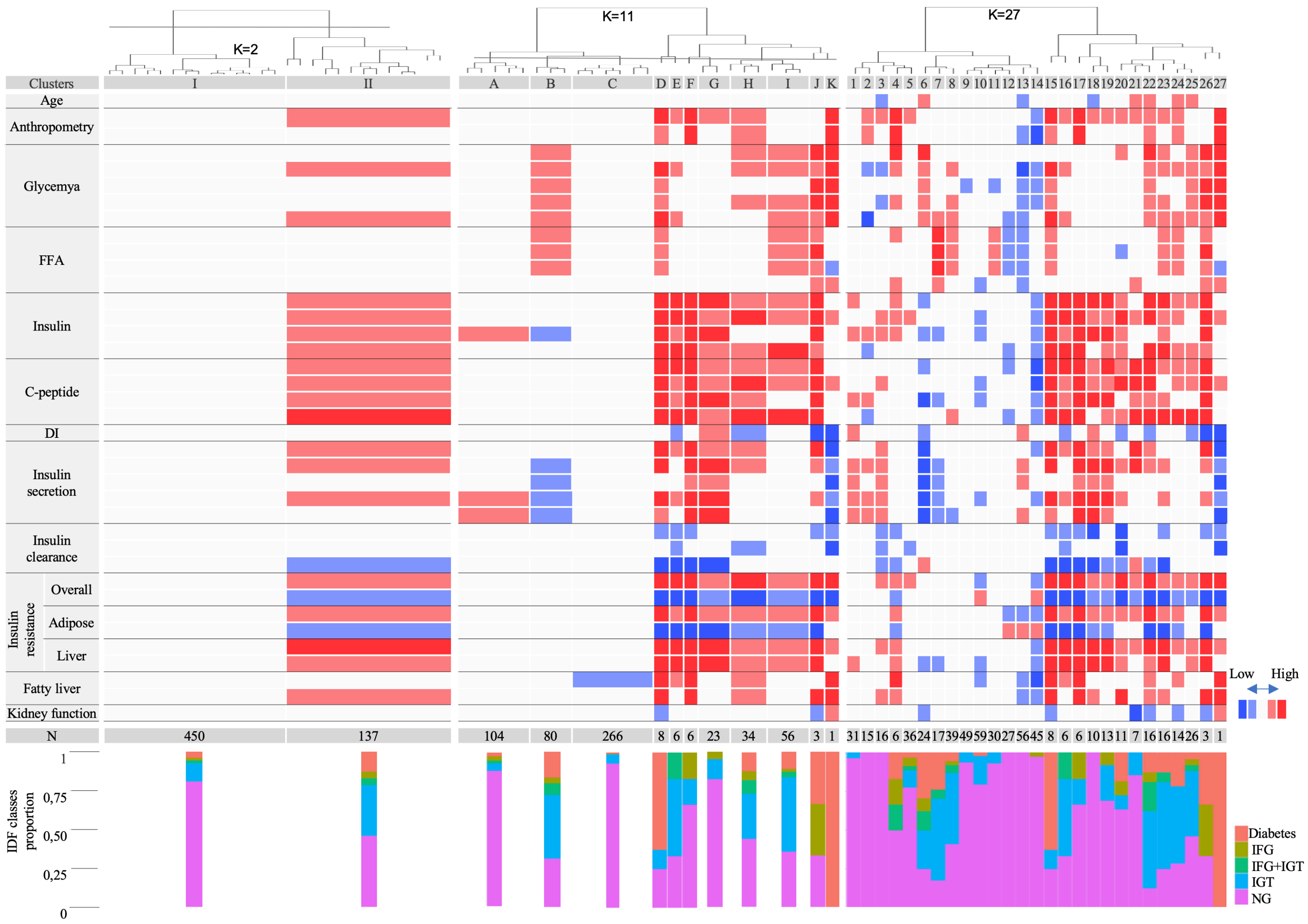

Supplementary Figure 3. Clusters profiling for the female population. Dendogram of the hierarchical clustering of the 27 Self Organizing Map (SOM) units is shown for three cut levels of the dendogram(a): K=2 (clusters I and II); K=11 (clusters A-K) and K=27 (clusters 1-27). For each cut level, the clusters were profiled with several parameters, herein grouped by function, and the median of each cluster was compared with the distribution of the overall population (percentiles 10,25,75,90), and are displayed with a heatmap scale (b). Number of subjects in each cluster is shown in (c). Distribution of IDF dysglycemia classes in each cluster is shown in (d). FFA, free fatty acids; DI, Disposition Index.

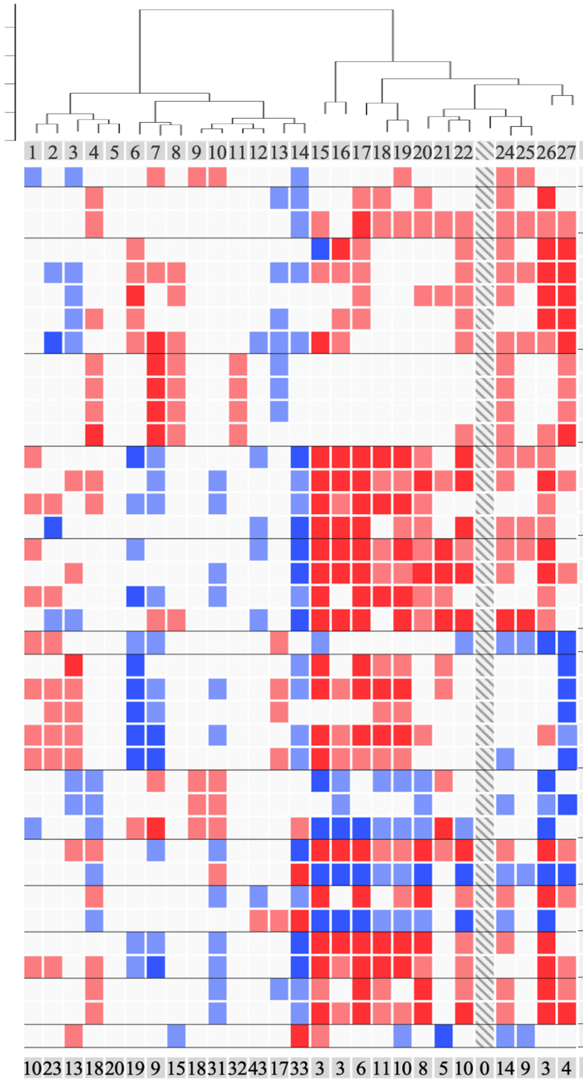

Low ← → High

Clusters	Heat map cut-offs				
	<	[, [	[, [	[, [	≥
**Age, years**	**42**	**42 , 53**	**53 , 70**	**70 , 77**	**77**
**BMI, kg/m²**	**22**	**22 , 24**	**24 , 30**	**30 , 33**	**33**
**Waist, cm**	**84**	**84 , 90**	**90 , 102**	**102 , 109**	**109**
HbA1c, %	5.0	5.0 , 5.2	5.2 , 5.8	5.8 , 6.0	6.0
$AUC_{(0\text{-}30\text{-}120)}$Glucose	**731**	**731 , 823**	**823 , 1067**	**1067 , 1223**	**1223**
**Fasting glucose, mmol/L**	**4.74**	**4.74 ,5.06**	**5.06 , 6.00**	**6.00 , 6.58**	**6.58**
**Glucose 30 min, mmol/L**	**6.91**	**6.91 , 8.00**	**8.00 , 10.53**	**10.53 , 11.81**	**11.81**
**Glucose 120 min, mmol/L**	**4.42**	**4.42 , 5.35**	**5.35 , 8.12**	**8.12 , 10.17**	**10.17**
$AUC_{(0\text{-}30\text{-}120)}$ FFA	18.5	18.5 , 24.8	24.8 , 44.4	44.4 , 57.0	57.0
**Fasting FFA, mmol/L**	**0.27**	**0.27 , 0.36**	**0.36 , 0.63**	**0.63 , 0.77**	**0.77**
**FFA 30 min, mmol/L**	**0.18**	**0.18 , 0.25**	**0.22 , 0.47**	**0.47 , 0.60**	**0.60**
**FFA 120 min, mmol/L**	**0.06**	**0.06 , 0.08**	**0.08 , 0.18**	**0.18 , 0.24**	**0.24**
$AUC_{(0\text{-}30\text{-}120)}$Insulin	**15995**	**15995 , 21544**	**21544 ,44729**	**44729 , 68193**	**68193**
**Fasting insulin, pmol/L**	**21.8**	**21.8 , 29.0**	**29.0 , 68.6**	**68.6 , 98.4**	**98.4**
**Insulin 30 min, pmol/L**	**144.9**	**144.9 , 208.3**	**208.3 , 466.7**	**466.7 , 696.1**	**696.1**
**Insulin 120 min, pmol/L**	**75.0**	**75.0 , 134.6**	**134.6 , 359.0**	**359.0 , 652.0**	**652.0**
$AUC_{(0\text{-}30\text{-}120)}$C-peptide	**165**	**165 , 206**	**206 , 316**	**316 , 393**	**393**
**Fasting C-peptide, nmol/L**	**0.43**	**0.43 , 0.54**	**0.54 , 0.88**	**0.88 , 1.13**	**1.13**
**C-peptide 30 min, nmol/L**	**1.25**	**1.25 , 1.65**	**1.65 , 2.65**	**2.65 , 3.31**	**3.31**
**C-peptide 120 min, nmol/L**	**1.38**	**1.38 , 1.84**	**1.84 , 3.08**	**3.08 , 4.00**	**4.00**
Disposition Index, $mmol^{-1}$	0.60	0.60 , 0.96	0.96 , 2.77	2.77 , 4.72	4.72
HOMA-B2	83	83 , 101	101 , 148	148 , 179	179
$AUC_{(0\text{-}30)}$C-peptide/$AUC_{(0\text{-}30)}$Glucose	0.12	0.12 , 0.15	0.15 , 0.24	0.24 , 0.30	0.30
$\Delta_{(0\text{-}30)}$C-peptide/$\Delta_{(0\text{-}30)}$Glucose	0.17	0.17 , 0.26	0.26 , 0.57	0.57 , 0.89	0.89
$AUC_{(0\text{-}30)}$Insulin/$AUC_{(0\text{-}30)}$Glucose	11.7	11.7 , 16.4	16.4 , 36.7	36.7 , 52.2	52.17
IGI	27.8	27.8 , 43.9	43.9 , 128.3	128.3 , 210.8	210.75
Fasting IC (10^{-3})	9.6	9.6 , 11.8	11.8 , 19.9	19.9 , 24.2	24.16
$\Delta_{(0\text{-}30)}$IC (10^{-4})	1.06	1.06 , 1.65	1.65 , 3.58	3.58 , 4.50	4.50
IC 120 min (10^{-3})	4.17	4.17 , 5.21	5.21 , 8.50	8.50 , 10.35	10.35
HOMA-IR2	0.40	0.40 , 0.54	0.54 , 1.30	1.30 , 1.90	1.90
Matsuda Index	2.49	2.49 , 3.96	3.96 , 8.95	8.95 , 12.40	12.40
Adipo-IR	8.3	8.3 , 12.8	12.8 , 35.3	35.3 , 54.4	54.4
ISI-FFA	0.75	0.75 , 0.98	0.98 , 1.5	1.5 , 1.7	1.7
Liver-IR	1.63	1.63 , 1.71	1.71 , 1.91	1.91 , 2.00	2.00
Hepatic-IR (10^5)	5.49	5.49 , 7.83	7.83 , 18.07	18.07 , 27.77	27.77
FLI	14	14-27	27 , 70	70 , 86	86
NAFLD-FLS	-2.73	-2.73 , -2,21	-2.21, -0.40	-0.40 , 0.55	0.55
eGFR (ml/min/1.73m²)	58	58 , 67	67 , 87	87 , 96	96
N					

Supplementary Figure 4. Clusters profiling for K=27, males. Clusters were profiled with several parameters besides the ones that inform the cluster analysis (bold) and the median of each cluster was compared with the distribution of the overall population (percentiles 10,25,75,90), and are displayed with a heatmap scale. The colors are defined by comparing the cluster median of the parameters with the distribution of the overall population (10th, 25th, 75th and 90th percentiles). The table on the right shows the quantile limit values for each profiling parameter. Number of subjects in each cluster is displayed below the heatmap (N). AUC, area under the curve; FFA, free fatty acids; IC, Insulin Clearance.

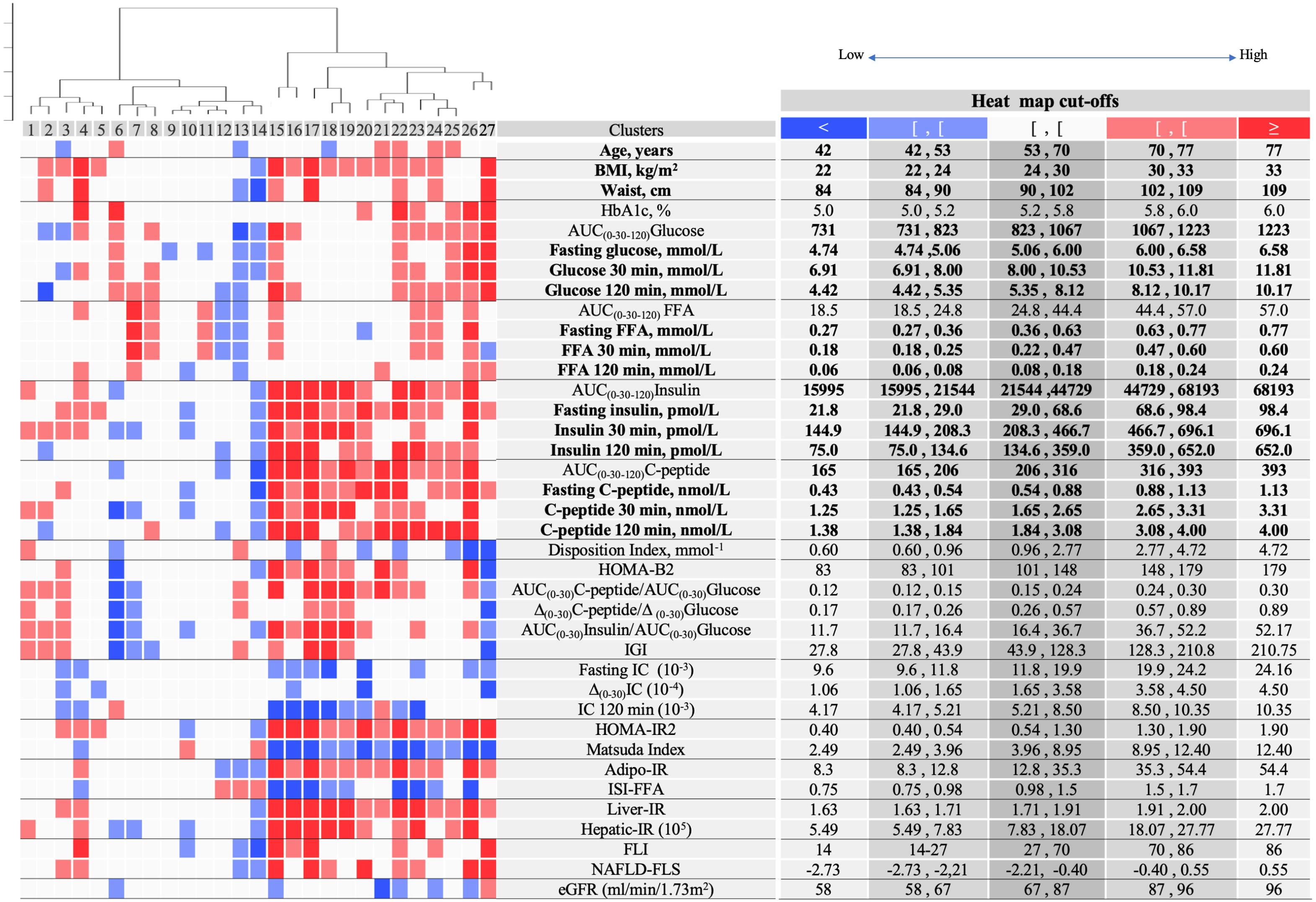

Low ⟷ High

Clusters	Heat map cut-offs				
	<	[, [	[, [	[, [	≥
**Age, years**	**42**	**42 , 53**	**53 , 70**	**70 , 77**	**77**
**BMI, kg/m²**	**22**	**22 , 24**	**24 , 30**	**30 , 33**	**33**
**Waist, cm**	**84**	**84 , 90**	**90 , 102**	**102 , 109**	**109**
HbA1c, %	5.0	5.0 , 5.2	5.2 , 5.8	5.8 , 6.0	6.0
$AUC_{(0\text{-}30\text{-}120)}$Glucose	**731**	**731 , 823**	**823 , 1067**	**1067 , 1223**	**1223**
**Fasting glucose, mmol/L**	**4.74**	**4.74 ,5.06**	**5.06 , 6.00**	**6.00 , 6.58**	**6.58**
**Glucose 30 min, mmol/L**	**6.91**	**6.91 , 8.00**	**8.00 , 10.53**	**10.53 , 11.81**	**11.81**
**Glucose 120 min, mmol/L**	**4.42**	**4.42 , 5.35**	**5.35 , 8.12**	**8.12 , 10.17**	**10.17**
$AUC_{(0\text{-}30\text{-}120)}$ FFA	18.5	18.5 , 24.8	24.8 , 44.4	44.4 , 57.0	57.0
**Fasting FFA, mmol/L**	**0.27**	**0.27 , 0.36**	**0.36 , 0.63**	**0.63 , 0.77**	**0.77**
**FFA 30 min, mmol/L**	**0.18**	**0.18 , 0.25**	**0.22 , 0.47**	**0.47 , 0.60**	**0.60**
**FFA 120 min, mmol/L**	**0.06**	**0.06 , 0.08**	**0.08 , 0.18**	**0.18 , 0.24**	**0.24**
$AUC_{(0\text{-}30\text{-}120)}$Insulin	**15995**	**15995 , 21544**	**21544 ,44729**	**44729 , 68193**	**68193**
**Fasting insulin, pmol/L**	**21.8**	**21.8 , 29.0**	**29.0 , 68.6**	**68.6 , 98.4**	**98.4**
**Insulin 30 min, pmol/L**	**144.9**	**144.9 , 208.3**	**208.3 , 466.7**	**466.7 , 696.1**	**696.1**
**Insulin 120 min, pmol/L**	**75.0**	**75.0 , 134.6**	**134.6 , 359.0**	**359.0 , 652.0**	**652.0**
$AUC_{(0\text{-}30\text{-}120)}$C-peptide	**165**	**165 , 206**	**206 , 316**	**316 , 393**	**393**
**Fasting C-peptide, nmol/L**	**0.43**	**0.43 , 0.54**	**0.54 , 0.88**	**0.88 , 1.13**	**1.13**
**C-peptide 30 min, nmol/L**	**1.25**	**1.25 , 1.65**	**1.65 , 2.65**	**2.65 , 3.31**	**3.31**
**C-peptide 120 min, nmol/L**	**1.38**	**1.38 , 1.84**	**1.84 , 3.08**	**3.08 , 4.00**	**4.00**
Disposition Index, $mmol^{-1}$	0.60	0.60 , 0.96	0.96 , 2.77	2.77 , 4.72	4.72
HOMA-B2	83	83 , 101	101 , 148	148 , 179	179
$AUC_{(0\text{-}30)}$C-peptide/$AUC_{(0\text{-}30)}$Glucose	0.12	0.12 , 0.15	0.15 , 0.24	0.24 , 0.30	0.30
$\Delta_{(0\text{-}30)}$C-peptide/$\Delta_{(0\text{-}30)}$Glucose	0.17	0.17 , 0.26	0.26 , 0.57	0.57 , 0.89	0.89
$AUC_{(0\text{-}30)}$Insulin/$AUC_{(0\text{-}30)}$Glucose	11.7	11.7 , 16.4	16.4 , 36.7	36.7 , 52.2	52.17
IGI	27.8	27.8 , 43.9	43.9 , 128.3	128.3 , 210.8	210.75
Fasting IC (10^{-3})	9.6	9.6 , 11.8	11.8 , 19.9	19.9 , 24.2	24.16
$\Delta_{(0\text{-}30)}$IC (10^{-4})	1.06	1.06 , 1.65	1.65 , 3.58	3.58 , 4.50	4.50
IC 120 min (10^{-3})	4.17	4.17 , 5.21	5.21 , 8.50	8.50 , 10.35	10.35
HOMA-IR2	0.40	0.40 , 0.54	0.54 , 1.30	1.30 , 1.90	1.90
Matsuda Index	2.49	2.49 , 3.96	3.96 , 8.95	8.95 , 12.40	12.40
Adipo-IR	8.3	8.3 , 12.8	12.8 , 35.3	35.3 , 54.4	54.4
ISI-FFA	0.75	0.75 , 0.98	0.98 , 1.5	1.5 , 1.7	1.7
Liver-IR	1.63	1.63 , 1.71	1.71 , 1.91	1.91 , 2.00	2.00
Hepatic-IR (10^{5})	5.49	5.49 , 7.83	7.83 , 18.07	18.07 , 27.77	27.77
FLI	14	14-27	27 , 70	70 , 86	86
NAFLD-FLS	-2.73	-2.73 , -2,21	-2.21, -0.40	-0.40 , 0.55	0.55
eGFR (ml/min/1.73m²)	58	58 , 67	67 , 87	87 , 96	96

Supplementary Figure 6. Clusters profiling for K=27, females. Clusters were profiled with several parameters besides the ones that inform the cluster analysis (bold) and the median of each cluster was compared with the distribution of the overall population (percentiles 10,25,75,90), and are displayed with a heatmap scale. The colors are defined by comparing the cluster median of the parameters with the distribution of the overall population (10th, 25th, 75th and 90th percentiles). The table on the right shows the quantile limit values for each profiling parameter. Number of subjects in each cluster is displayed below the heatmap (N). AUC, area under the curve; FFA, free fatty acids; IC, Insulin Clearance.

Parameter	Formula and calculation	Reference
$AUC_{(0-30-120)}$Glucose	Trapezoidal rule	-
$AUC_{(0-30-120)}$FFA	Trapezoidal rule	-
$AUC_{(0-30-120)}$Insulin	Trapezoidal rule	-
$AUC_{(0-30-120)}$C-peptide	Trapezoidal rule	-
HOMA-B2	HOMA calculator (University of Oxford, Oxford, UK, using C-peptide	(21)
$AUC_{(0-30)}$C-peptide/$AUC_{(0-30)}$Glucose	(C-peptide0min+C-peptide30min)/(Glucose0min+Glucose30min)	-
$AUC_{(0-30)}$Insulin/$AUC_{(0-30)}$Glucose	(Insulin0min+Insulin30min)/(Glucose0min+Glucose30min)	-
Insulinogenic index (IGI)	(Insulin30min–Insulin0min)/(Glucose30min-Glucose0min)	-
Disposition Index (DI)	IGI/Insulin0min	(22)
HOMA_IR2	HOMA calculator (University of Oxford, Oxford, UK, using Insulin	(21)
Matsuda Index	Calculated with three time points (0, 30 and 120 minutes) of the OGTT	(22)
Liver-IR Index	-0.091+(log $AUC_{(0-120)}$Insulin x0.400)+(logfatmass% x0.346)–(logHDLx0.408)+(logBMIx0.435)	(23)
Hepatic-IR Index	$AUC_{(0-30)}$Insulin x $AUC_{(0-30)}$Glucose	(24)
Adipo-IR Index	FFA0min*Insulin0min	(25)
ISI-FFA Index	2/[($AUC_{(0-30-120)}$Insulin x ($AUC_{(0-30-120)}$FFA) + 1]	(26)
Fasting Insulin Clearance (IC)	C-peptide0min/Insulin0min	-
IC at 30 minutes of the OGTT	$AUC_{(0-30)}$C-peptide/$AUC_{(0-30)}$Insulin	-
IC at 120 minutes of the OGTT	$AUC_{(30-120)}$C-peptide/$AUC_{(30-120)}$Insulin	-
$\Delta_{(0-30)}$IC	slope of insulin clearance at fast and insulin clearance at 30min	-
Non-alcoholic Fatty Liver Disease – Fatty Liver Score (NAFLD-FLS)	2.889+1.179xIDFmetabolic syndrome(yes=1/no=0)+0.454xtype2diabetes(yes=2/no=0)+0.145xInsulin 0min(mIU/l)	(28)
Fatty liver Index (FLI)	$e^{0.953xln(TG)+0.139xBMI+0.053xWaist\ circumference-15.745}$)/(1+$e^{0.953xln(TG)+0.139xBMI+0.053xWaist\ circumference-15.745}$)x100	(29)
BMI	weight (kg)/height^2(m)	-
eGFR	CKD-EPI formula	(30)

Supplementary Table1– Indexes formulas and calculations of the parameters used to profile the clusters.
